# Can using a pre-trained deep learning model as the feature extractor in the bag-of-deep-visual-words model always improve image classification accuracy?

**DOI:** 10.1371/journal.pone.0298228

**Published:** 2024-02-29

**Authors:** Ye Xu, Xin Zhang, Chongpeng Huang, Xiaorong Qiu

**Affiliations:** School of IoT Technology, Wuxi Institute of Technology, Wuxi, Jiangsu, China; University of California Los Angeles, UNITED STATES

## Abstract

This article investigates whether higher classification accuracy can always be achieved by utilizing a pre-trained deep learning model as the feature extractor in the Bag-of-Deep-Visual-Words (BoDVW) classification model, as opposed to directly using the new classification layer of the pre-trained model for classification. Considering the multiple factors related to the feature extractor -such as model architecture, fine-tuning strategy, number of training samples, feature extraction method, and feature encoding method—we investigate these factors through experiments and then provide detailed answers to the question. In our experiments, we use five feature encoding methods: hard-voting, soft-voting, locally constrained linear coding, super vector coding, and fisher vector (FV). We also employ two popular feature extraction methods: one (denoted as Ext-DFs(CP)) uses a convolutional or non-global pooling layer, and another (denoted as Ext-DFs(FC)) uses a fully-connected or global pooling layer. Three pre-trained models—VGGNet-16, ResNext-50(32×4d), and Swin-B—are utilized as feature extractors. Experimental results on six datasets (15-Scenes, TF-Flowers, MIT Indoor-67, COVID-19 CXR, NWPU-RESISC45, and Caltech-101) reveal that compared to using the pre-trained model with only the new classification layer re-trained for classification, employing it as the feature extractor in the BoDVW model improves the accuracy in 35 out of 36 experiments when using FV. With Ext-DFs(CP), the accuracy increases by 0.13% to 8.43% (averaged at 3.11%), and with Ext-DFs(FC), it increases by 1.06% to 14.63% (averaged at 5.66%). Furthermore, when all layers of the pre-trained model are fine-tuned and used as the feature extractor, the results vary depending on the methods used. If FV and Ext-DFs(FC) are used, the accuracy increases by 0.21% to 5.65% (averaged at 1.58%) in 14 out of 18 experiments. Our results suggest that while using a pre-trained deep learning model as the feature extractor does not always improve classification accuracy, it holds great potential as an accuracy improvement technique.

## 1 Introduction

Deep Learning (DL) technology has made significant strides in various computer vision tasks such as image classification [1, 2], object detection [3, 4], semantic segmentation [5], and pedestrian detection [6], among others. With the support of ample training samples and robust computing resources, deep neural network architectures can discern complex patterns hidden within these samples. However, the acquisition of a large number of training samples can be costly for many tasks. To address this challenge, many researchers have turned to DL models pre-trained on other large-scale datasets to effectively reduce the need for extensive training samples.

Pre-trained DL models can be used in five main ways for image classification tasks [7]. The first method involves fine-tuning a pre-trained DL model with a limited number of training samples from a new task [8, 9]. The second approach focus on extracting image patches and converting them into deep features using a DL model, following the Bag-of-Visual-Words (BoVW) model workflow [10–13]. The third method [14, 15] enhances the second by generating high-semantic image patches using object detection models (e.g., Faster RNN [16], SSD [17], YOLO [18]). The fourth approach leverages features from different layers of a DL model [19, 20], considering that low-layer features capture small objects with precise location but less semantics, while high-layer features capture larger objects with rich semantics. The fifth approach integrates multiple features from complementary DL models to represent complex scenes, capitalizing on the fact that features generated from models trained on different datasets are usually complementary [21, 22].

Among these, the second method, known as the Bag-of-Deep-Visual-Words (BoDVW) image classification model, is notable for its simplicity and effectiveness [23–25]. Unlike the BoVW model, which calculates handcrafted features over small regions, the BoDVW model extracts deep features using a pre-trained DL model. These deep features carry more semantic information and larger receptive fields. The remaining stages, including dictionary learning, feature encoding, and feature pooling, are identical to those of the BoVW model, allowing methodologies from the BoVW era to be applied to the BoDVW model [26–28].

Recently, the BoDVW model has been employed to tackle a variety of image classification tasks. Sitaula et al. [25] proposed a classification method for chest X-Ray images based on the BoDVW model. Their approach enhanced the accuracy by approximately 15% and 3% compared to [29, 30] respectively, which were sophisticatedly designed convolutional neural networks (CNNs) introduced in 2020. Saini et al. [12] utilized the BoDVW model to address the imbalance issue encountered when processing multi-class image datasets. In the realm of remote sensing classification tasks, the BoDVW model’s classification accuracy surpasses CNN-based methods by about 6% [23]. Xie et al. [20] and Pour et al. [31] have applied the BoDVW model for scene recognition and ship classification tasks, respectively.

Although the aforementioned studies have applied the BoDVW model, it remains unclear whether higher classification accuracy can always be achieved by employing a pre-trained DL model as the feature extractor in the BoDVW model, compared to directly using the new classification layer of the pre-trained model for classification. In other words, can the use of a pre-trained DL model as the feature extractor in the BoDVW model always improve image classification accuracy? Given that there are multiple factors closely related to the feature extractor, including model architecture, fine-tuning strategy, number of training samples, feature extraction method, and feature encoding method, we conducted a detailed investigation into these factors. We then answered this key question based on our experimental results.

Firstly, we investigate the factor: feature encoding method. Various coding methods have been proposed in the era of the BoVW model, such as hard-voting (HV) [32], soft-voting (SV) [33], sparse coding [34], local-constrained linear coding (LLC) [35], laplacian sparse coding [36], super vector coding (SVC) [37], fisher vector (FV) [38], and more. Some of these methods have found their application in the BoDVW model. For example, [12] adopted HV, sparse coding was the method of choice in [39], LLC was implemented in [40], while [11] opted for FV. However, these haven’t been compared fairly when the coding objects are deep features. Therefore, the first step in our research is to conduct a comprehensive comparison of five representative encoding methods (HV, SV, LLC, SVC, and FV).

Next, we examine another factor: feature extraction method. Existing literature presents two DL model-based feature extraction methods. One method (denoted as Ext-DFs(CP)) extracts deep features from the feature map of a convolutional or non-global pooling layer. The other (denoted as Ext-DFs(FC)) extracts multi-scale image patches and uses the output vectors from a fully-connected or global pooling layer as deep features. A recent study [41] utilized these two methods, but it did not provide experimental results under different dictionary sizes and encoding methods, and also did not fully analyze the computational costs. In this article, we take a closer look at these two methods.

Having discussed the above two factors, we turn to the remaining factors: model architecture, fine-tuning strategy, and number of training samples. We employ three pre-trained DL models with different architectures as feature extractors: VGGNet-16 [42], ResNext-50(32×4d) (hereafter referred to as ResNext-50) [43], and Swin-B (denoted as SwinTransformer) [44]. Two fine-tuning strategies FT(part) and FT(all) are adopted. FT(part) only retrains the new classification layer of pre-trained DL models, while FT(all) fine-tunes all layers of models. Different numbers of training samples are used to fine-tune pre-trained DL models. Here, we chose only FV as the feature encoding method, because in the comparative experiment of feature encoding, FV showed higher accuracy compared to other encoding methods with low computational cost.

Our experimental results on six diverse datasets (15-Scenes [45], TF-Flowers [46], MIT Indoor-67 [47], COVID-19 CXR [48], NWPU-RESISC45 [49], and Caltech-101 [50]) indicate that, while the practice of using a pre-trained DL model as the feature extractor in the BoDVW model may not invariably enhance the classification accuracy, it exhibits significant potential as an improvement technique. Specifically, in comparison to the classification accuracy achieved by the DL model fine-tuned by FT(part), this practice improves the classification accuracy in 35 out of 36 experiments when using FV. The accuracy increases by 0.13% to 8.43% (averaged at 3.11%) with Ext-DFs(CP) and by 1.06% to 14.63% (averaged at 5.66%) with Ext-DFs(FC). Moreover, when the DL model is fine-tuned by FT(all) and employed as the feature extractor, this practice results in accuracy gains in 14 out of 18 experiments by 0.21% to 5.65% (averaged at 1.58%), if FV and Ext-DFs(FC) are used.

Our work makes two key contributions. First, we conduct extensive experiments to investigate the potential of using a pre-trained DL model as the feature extractor to improve classification accuracy. The extent of accuracy improvement brought about by this practice is elucidated under different hyperparameter subspaces. Second, we analysis the five factors investigated in our experiments from the perspective of high-level semantics based on our experimental results.

The structure of this article is as follows: the subsequent section discusses related work. Section 3 outlines the methodology. Section 4 delves into the experimental evaluation and analysis, and the article concludes with Section 5. For clarity, Table 1 summarizes the abbreviations and their meanings used in this article.

**Table 1:** Abbreviates and their meanings.

Abbreviate	Meaning
DL	deep learning
HV	hard-voting
SV	soft-voting
LLC	locality-constrained linear coding
SVC	super vector coding
FV	fisher vector
Ext-DFs(CP)	extracting deep features via a convolutional or non-global pooling layer
Ext-DFs(FC)	extracting deep features via a fully-connected or global pooling layer
FT(part)	only the new classification layer is retrained
FT(all)	all the layers are fine-tuned

## 2 Related work

**Fig 1:**
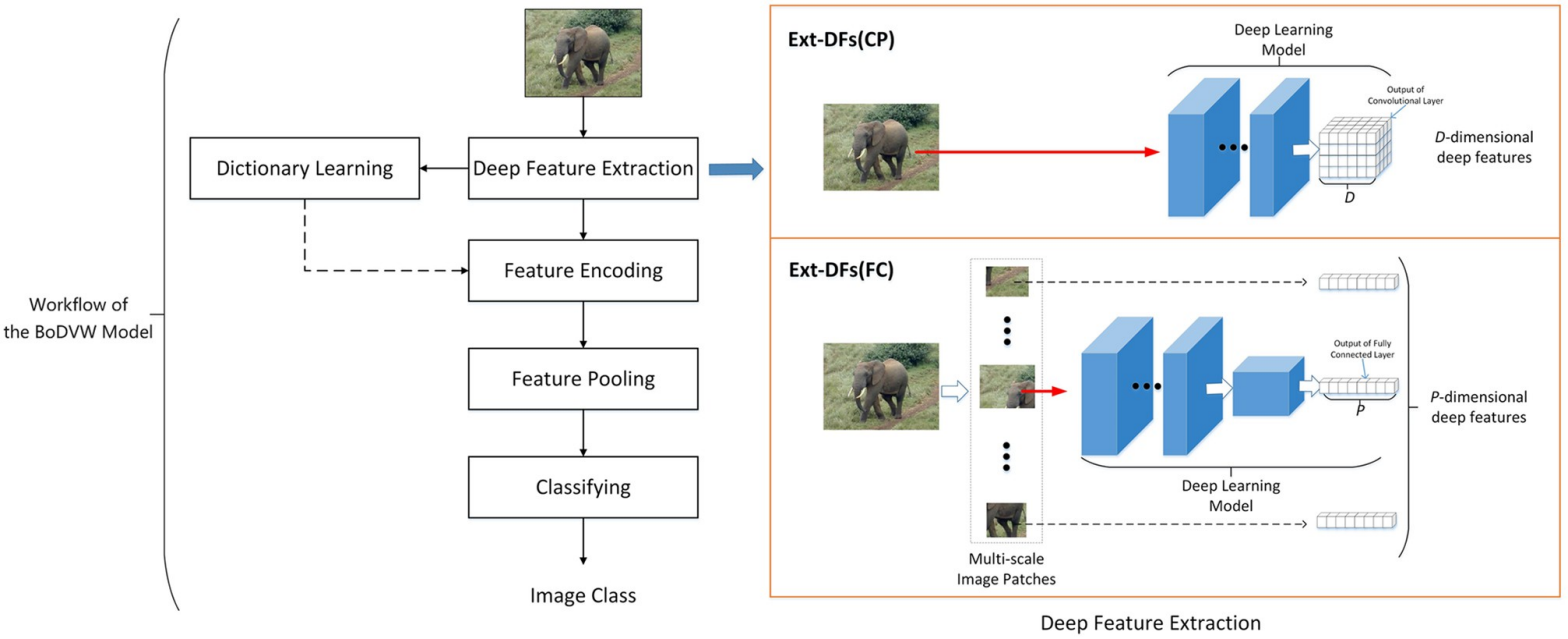
Workflow of the BoDVW model.

The BoDVW model integrates the traditional BoVW model with the DL model. As shown in Fig 1, initially, the BoDVW model employs a pre-trained DL model to extract deep features from the image. It then applies the dictionary learning, feature encoding, and feature pooling steps from the BoVW model to generate a representation vector for the image, which is subsequently used for image classification. In comparison to handcrafted features used in the BoVW model, deep features extracted by the BoDVW model encompass higher-level semantic information and larger receptive fields. As a result, the BoDVW model shows superior performance across a variety of image classification tasks compared to the BoVW model. In the following, we review the related work on feature extractor, feature extraction, and feature encoding.

### 2.1 Feature extractor

The BoDVW model utilizes a pre-trained DL model to extract deep features. Various pre-trained models have already been employed as feature extractors in existing works. For instance, AlexNet, GoogleNet, and VGGNet-16 were used in [23], ResNet-50 was utilized in [12], and AlexNet and DeCAF_6_ were respectively employed in [20, 40].

Considering the evolution of DL model architectures over the past decade, this article briefly reviews three representative models: VGGNet-16, ResNext-50(32 × 4d), and Swin-B, which are all used in this article. The architectures of the three DL models are detailed in Table 2.

**Table 2:** Configurations of the model architectures of VGGNet-16, ResNext-50 (32x4d), and Swin-B. In the notation [kernel size × kernel size, channels;…] × *N*, *N* denotes the number of times a sequence of convolution layers is repeated, with each layer in the sequence defined by its kernel size and output channels. ‘C’ refers to the cardinality (number of groups). ‘concat *n* × *n*’ indicates downsampling by concatenating *n* × *n* neighboring features. ‘LN’ abbreviates LayerNorm. ‘win. sz. *n* × *n*’ refers to a multi-head self-attention module with a window size of *n* × *n*. ‘*D*-d’ signifies a linear layer with an output dimension of *D*. △ and ⋆ denote Ext-DFs(CP) and Ext-DFs(FC) respectively. The output of the cells where these symbols are located is utilized as the source of deep features.

VGGNet-16	RexNext-50(32×4d)	Swin-B
[3×3, 64]×2	7×7,64, stride 2	concat 4×4, 128-d, LN
maxpool	3×3 max pool, stride 2	$[\begin{array}{c} \text{win.}\;\text{sz.}\;7\times 7,\\ \text{dim}\;\text{128,}\;\text{head}\;\text{4} \end{array}]\times 2$
[3×3, 128]×2	$[\begin{array}{c} 1\times 1,128\\ 3\times 3,128,C=32\\ 1\times 1,256 \end{array}]\times 3$	concat 2×2, 256-d, LN
maxpool	$[\begin{array}{c} 1\times 1,256\\ 3\times 3,256,C=32\\ 1\times 1,512 \end{array}]\times 4$	$[\begin{array}{c} \text{win}.\;\text{sz}.\;7\times 7,\\ \text{dim}\;256,\;\text{head}\;8 \end{array}]\times 2$
[3×3, 256]×21×1,256	$[\begin{array}{c} 1\times 1,512\\ 3\times 3,512,\text{C}=32\\ 1\times 1,1024 \end{array}]\times 2$	concat 2×2, 512-d, LN
maxpool	△ $[\begin{array}{c} 1\times 1,1024\\ 3\times 3,1024,\text{C}=32\\ 1\times 1,2048 \end{array}]\times 3$	$[\begin{array}{c} \text{win}.\;\text{sz}.\;7\times 7,\\ \text{dim}\;512,\;\text{head}\;16 \end{array}]\times 18$
[3×3, 512]×21×1,512	⋆ global average pool	concat 2×2, 1024-d, LN
maxpool	1000-d	$[\begin{array}{c} \text{win}.\;\text{sz}.\;7\times 7,\\ \text{dim}\;1024,\;\text{head}\;32 \end{array}]\times 2$
△ [3×3, 512]×21×1,512	softmax	△ LN
maxpool		⋆ global average pool
⋆ [4096-d]×2		1000-d
1000-d		softmax
softmax		

VGGNet-16 is a member of the VGGNet family. The VGGNet family is characterized by its use of small 3×3 convolutional filters and 2×2 max pooling kernels, which are stacked to increase the depth of the network while maintaining a manageable number of parameters. This design allows VGGNet models to excel in capturing fine-grained image details. The VGGNet family includes several models, with VGGNet-16 and VGGNet-19 being the most well-known. VGGNet-16, consisting of 13 convolutional layers and 3 fully connected layers, is a representative model in the VGGNet family.

ResNext-50(32×4d) is a variant of the ResNet family, which introduced the concept of residual connections to alleviate the problem of vanishing gradients in deep networks. The ResNet family is known for its repeating block structure, where each block consists of a bottleneck design with three layers: a 1×1 convolutional layer, a 3×3 convolutional layer, and another 1×1 convolutional layer. ResNext-50 extends this architecture by introducing grouped convolutions, which increase the model’s capacity without a significant increase in computational complexity. ResNext-50 is a representative model in the ResNet family that balances performance and computational efficiency.

Swin-B is a variant of the SwinTransformer family, a new class of models that apply transformer architectures to computer vision tasks. Unlike traditional convolutional models, SwinTransformer models divide the input image into non-overlapping patches and process them with self-attention mechanisms, allowing them to capture long-range dependencies within the image. The SwinTransformer family includes several configurations with different numbers of layers, hidden sizes, and heads. Swin-B is a representative model in the SwinTransformer family, offering a good balance between performance and computational efficiency. Notably, Swin-B is a general-purpose model that was designed for a variety of vision tasks, not just image classification, the model archietecture listed in Table 2 has been adapted for image classification and is consistent with the official PyTorch implementation.

### 2.2 Feature extraction

Feature extraction is a critical step in the BoDVW model, which involves using a pre-trained DL model to extract high-semantic features from images. Two primary methods of feature extraction have been identified in the literature, as shown in Fig 1.

The first method, referred to as Ext-DFs(CP), directly inputs an image into a pre-trained DL model, and the feature map of a convolutional or non-global pooling layer is used as the source of deep features. For a feature map with dimensions *W*×*H*×*D*, where *D* represents the number of channels, this feature map can generate *W*×*H* *D*-dimensional deep features.

In terms of related work, the study [23] used the layer “conv5” of AlexNet, the layer “inception 4(e)” of GoogleNet, and the layer “conv5-3” of VGGNet-16 to extract deep features. These schemes generated 13×13 256-dimensional features, 14×14 832-dimensional features, and 14×14 512-dimensional features for an image, respectively. Another study [12] used the output of the last convolutional layer of ResNet-50 as the source of deep features, resulting in 7×7 2048-dimensional features for each image.

The second method, referred to as Ext-DFs(FC), uses the output vectors for image patches obtained at a fully-connected or global pooling layer as deep features. Image patches can be extracted at multiple scales in a dense or well-chosen manner. Each image patch is resized to a pre-trained DL model’s input size, then input into the model. The output vector from a fully-connected or global pooling layer of the model is then used as a deep feature. If *N* image patches are extracted from an image, then *N* deep features will be obtained.

In terms of related work, the study [20] sampled image patches at the multiple scales of 128×128, 96× 96 and 64 ×64 pixels with a step size of 32 pixels, and the output vector of a deep fully-connected layer of AlexNet for each patch was regarded as a deep feature. Discriminative patches were chosen from training images based on selective search and spectral clustering. Another study [40] used image patches of 256 ×256, 224×224, 192 ×192, 160×160, and 128×128 pixels, and a deep fully-connected layer of DeCAF_6_ was chosen to obtain deep features.

In summary, both methods have their unique advantages and have been used effectively in various studies. The main difference between the two extraction methods lies in how deep features are derived. Deep features extracted by Ext-DFs(FC) are derived from image patches of different scales and positions. In contrast, deep features extracted by Ext-DFs(CP) can be viewed as being converted from image patches of the same scale at different positions, given that the receptive fields of deep features are the same in scale but vary in focus point.

### 2.3 Feature encoding

In the BoDVW model, deep features are encoded into coding vectors. Various coding methods have been developed and applied in the context of the BoVW model, and some of these have been adapted for use in the BoDVW model due to their similar workflows.

Huang et al. [13] conducted a comprehensive study on the coding methods developed for the BoVW model in 2014. They categorized the existing coding methods into five groups based on their underlying principles: voting-based, fisher coding-based, reconstruction-based, local tangent-based, and saliency-based. Several of these methods have been successfully applied to the BoDVW model.

Voting-based methods, such as hard voting, have been applied in the BoDVW model. For instance, in [12], each deep feature is encoded by its nearest visual word, resulting in a 0-1 vector where the element of value 1 corresponds to the word. The words are the centroids generated by *K*-means on a set of deep features.

Reconstruction-based methods, such as sparse coding and locality-constrained linear coding (LLC), have also been used to encode deep features. Khan et al. [39] used sparse coding to encode deep features, which are the output vectors for mid-level image patches obtained at a deep fully-connected layer of an AlexNet-like model. In [40], the authors used LLC to encode the output vectors of a fully-connected layer for multi-scale image patches.

Fisher Vector (FV) is another method that has been used in the BoDVW model. Xie et al. [20] encoded two kinds of deep features extracted by AlexNet for scene recognition. They used FV to encode the convolutional features yielded by the last convolutional layer, and LLC to encode the output vectors of the fully-connected layer. Gao et al. [11] evaluated the impact of the number *K* of the Gaussian Mixture Model (GMM)’s components on classification accuracy for FV. Diba et al. [51] iteratively learned discriminative clusters and then used FV to encode deep features.

In conclusion, a variety of coding methods from the BoVW model era have been applied to the BoDVW model, including voting-based, reconstruction-based, and fisher coding-based methods. These methods have contributed significantly to the success of the BoDVW model in various image classification tasks.

## 3 Methodology

This article is dedicated to addressing the question of whether higher classification accuracy can always be achieved by employing a pre-trained DL model as the feature extractor in the BoDVW model. To answer this question, we conducted a series of experiments. This section will provide the details of the experiments, including the datasets used, the detailed configuration of the BoDVW model, and the performance metric.

### 3.1 Datasets

This article utilizes six public image datasets: 15-Scenes, TF-Flowers, MIT Indoor-67, COVID-19 CXR, NWPU-RESISC45, and Caltech-101. These datasets cover four distinct scenarios: object recognition, scene classification, biomedical image classification, and remote sensing image classification.

- **15-Scenes:** This scene recognition dataset contains 4485 images distributed over 15 categories, primarily consisting of outdoor scenes.
- **TF-Flowers:** This dataset includes images from five different flower categories: Daisies, Dandelions, Tulips, Roses, and Sunflowers.
- **MIT Indoor-67:** This indoor scene recognition dataset encompasses 67 indoor categories with a total of 15630 images.
- **COVID-19 CXR:** This dataset features chest X-ray images for COVID-19-positive cases, along with images of normal and viral pneumonia cases.
- **NWPU-RESISC45:** This remote sensing classification dataset comprises 31500 images divided into 45 scene categories.
- **Caltech-101:** This object recognition dataset includes 9,144 images across 100 categories and one background category.

### 3.2 Experimental design

#### 3.2.1 Dataset division

Each image dataset is partitioned into separate training and testing sets, ensuring no overlap between them. The distribution of training and testing images per category is shown in Table 3.

**Table 3:** Distribution of training and testing images per category for each dataset.

Dataset	Training Images	Testing Images
15-Scenes	100	100
TF-Flowers	450	150
COVID-19 CXR	450	150
NWPU-RESISC45	140	60
MIT Indoor-67	80	20
Caltech-101	30	At most 20

#### 3.2.2 BoDVW model configuration

The BoDVW model involves several stages. In the following, we detail the specific settings adopted in each stage.

- **Feature Extraction Stage:** Three DL models, VGGNet-16, ResNext-50, and SwinTransformer, pre-trained on the ImageNet-1k dataset, are employed. The selection of these models as feature extractors was driven by their unique architectural characteristics and proven performance in image classification tasks. VGGNet-16, known for its depth and simplicity, has demonstrated robustness to scale and translation of images. ResNext-50, with its cardinality dimension, offers a way to increase model capacity without a significant increase in computational complexity. SwinTransformer, a recent model, brings the advantages of Transformer architectures to computer vision, effectively handling long-range dependencies in images.
  All models are fine-tuned using two strategies. The first strategy, denoted as FT(part), trains only the parameters of the new classification layer, while the second strategy, denoted as FT(all), fine-tunes the parameters of all layers. The two strategies, FT(part) and FT(all), were chosen to explore the trade-off between computational efficiency and performance. FT(part) allows us to leverage the power of pre-trained models with less computational cost, while FT(all) provides an opportunity to fully adapt the models to our specific task, potentially improving performance at the cost of increased computation.
  The fine-tuning of the pre-trained DL models is performed using the stochastic gradient descent algorithm with a learning rate of 0.001 and momentum of 0.9. The learning rate decays every 7 epochs by a factor of 0.1. For each model, two scenarios are considered. In the first scenario, FT(part), the model is directly utilized to extract features and is trained for a total of 30 epochs when used for image classification via its new classification layer. In the second scenario, FT(all), the parameters of all layers of the model are first fine-tuned using training images, and then the fine-tuned model is leveraged to extract features. In this case, the model is trained for a total of 50 epochs.
  Deep features are generated using Ext-DFs(CP) and Ext-DFs(FC). For Ext-DFs(CP), existing works such as [12, 23] use the deepest convolutional layer to generate deep features for high accuracy. For Ext-DFs(FC), it has been observed in [40] that the more diverse the scale size of image patches, the higher the accuracy. Following these existing works, we set up Ext-DFs(CP) and Ext-DFs(FC) in the following way.
  - **Ext-DFs(CP):** All images are resized to 224×224×3 pixels to match the input size of the pre-trained DL models. The deepest convolutional layer of VGGNet-16, the last convolutional layer of ResNext-50, and the normalization layer of SwinTransformer are used to generate deep features. For clarity, these specific layers are identified with △ in Table 2.
  - **Ext-DFs(FC):** Each image is scaled to a minimum side length of 256 pixels. Then, multiple patches are extracted from each image at six scales (96×96, 128×128, 160×160, 192×192, 224×224, and 256×256 pixels) with a stride of 32 pixels. All image patches are resized to 224×224×3 pixels and fed into the pre-trained DL models. The penultimate fully-connected layer of VGGNet-16, the global average pooling layer of ResNext-50, and the global average pooling layer of SwinTransformer are selected to yield deep features. For clarity, these specific layers are identified with ⋆ in Table 2.
Table 4 shows the dimensions of deep features obtained using different pre-trained DL models by Ext-DFs(CP) and Ext-DFs(FC).
- **Dictionary Learning Stage:** Deep features extracted from training images are used to learn a dictionary, with GMM applied for FV and *K*-means for other coding methods.
- **Feature Encoding Stage:** HV, SV, LLC, SVC, and FV are employed to encode features. The number of words for encoding each feature is set to 5 for SV, LLC, and 20 for SVC.
- **Feature Pooling Stage:** A spatial pyramid with the resolutions of 1 × 1, 2 × 2, 4 × 4 is used to obtain a representation vector for each image, followed by a *l*_1.5_-normalization operation (*l*_2_-normalizing the square roots of the element values in the vector)
- **Classifier Training Stage:** When HV is used, Support Vector Machines (SVMs) with a Chi-2 kernel are trained. For other coding methods, linear SVMs are trained.

**Table 4:** Dimensions of deep features obtained using different pre-trained DL models by Ext-DFs(CP) and Ext-DFs(FC). (The names of the layer are in accordance with the naming conventions in the PyTorch implementations of the models).

Method	DL Model (Layer)	Dimensions
Ext-DFs(CP)	VGGNet-16 (features.42)	512
	ResNext-50 (layer4.2.relu)	2048
	SwinTransformer (norm)	1024
Ext-DFs(FP)	VGGNet-16 (classifier.5)	4096
	ResNext-50 (avgpool)	2048
	SwinTransformer (avgpool)	1024

### 3.3 Performance metric

The metric used to evaluate the classification performance is accuracy. Accuracy is defined as the proportion of correct predictions made by the model out of all predictions. It is calculated as follows:

$$\begin{array}{c} \text{Accuracy}=\frac{\text{Number}\;\text{of}\;\text{Correct}\;\text{Predictions}}{\text{Total}\;\text{Number}\;\text{of}\;\text{Predictions}} \end{array}$$ (1)

This metric provides a straightforward measure of the model’s performance on the classification task. The choice to use accuracy as the sole performance metric is justified for two main reasons. Firstly, the training and testing sets derived from each dataset are balanced, with the exception of the Caltech-101 dataset. In the case of the Caltech-101 dataset, a few classes have an uneven number of samples. However, to mitigate this issue, we specifically selected a maximum of 20 images per class for testing. Secondly, our goal in this study is to assess the overall performance of the BoDVW model across all classes, rather than focusing on its performance on specific classes. Accuracy, which provides a straightforward measure of all correct predictions out of all predictions, aligns better with this goal.

## 4 Experimental results and analysis

In this section, we conducted an experimental analysis of several factors closely related to the feature extractor. These factors include model architecture, fine-tuning strategy, number of training samples, feature extraction method, and feature encoding method. We selected common settings for these factors. The settings for these factors include three different model architectures, two fine-tuning strategies, six different sample number settings, two feature extraction methods, and five feature encoding methods. These factors constitute a hyperparameter space. Based on this space, we aim to answer the key question of our study: Does the practice of using a pre-trained DL model as the feature extractor in the BoDVW model always improve classification accuracy? If the answer is no, we will explore the hyperparameter subspaces where this practice works consistently.

For feature encoding method, we compare five feature encoding methods—HV, SV, LLC, SVC, and FV—across six different datasets in Section 4.1. For feature extraction method, we compare two methods, namely Ext-DFs(CP) and Ext-DFs(FC). The experimental results obtained under different dictionary sizes, encoding methods, and feature extractors are provided in Section 4.2.

For model architecture, fine-tuning strategy, and number of training samples, we combine different pre-trained DL models and fine-tuning strategies and test their performance under two feature extraction methods. Three pre-trained DL models (VGGNet-16, ResNext-50, and SwinTransformer) and two fine-tuning strategies (FT(part) and FT(all)) are employed. In addition to fine-tuning with a sufficient number of training images (shown in Table 3), we also fine-tune the pre-trained SwinTransformer with an insufficient number of training images (e.g., 5 images per category). The experimental results of these three factors are discussed separately in Sections 4.3 to 4.5.

The detailed answer to the key question and an in-depth analysis of the five factors are provided in Section 4.6.

In the investigation of these factors, the accuracies achieved by all pre-trained DL models through their own classification layers serve as our **baselines**. This allows us to better understand and evaluate our experimental results.

### 4.1 Factor 1: Feature encoding method

**Fig 2:**
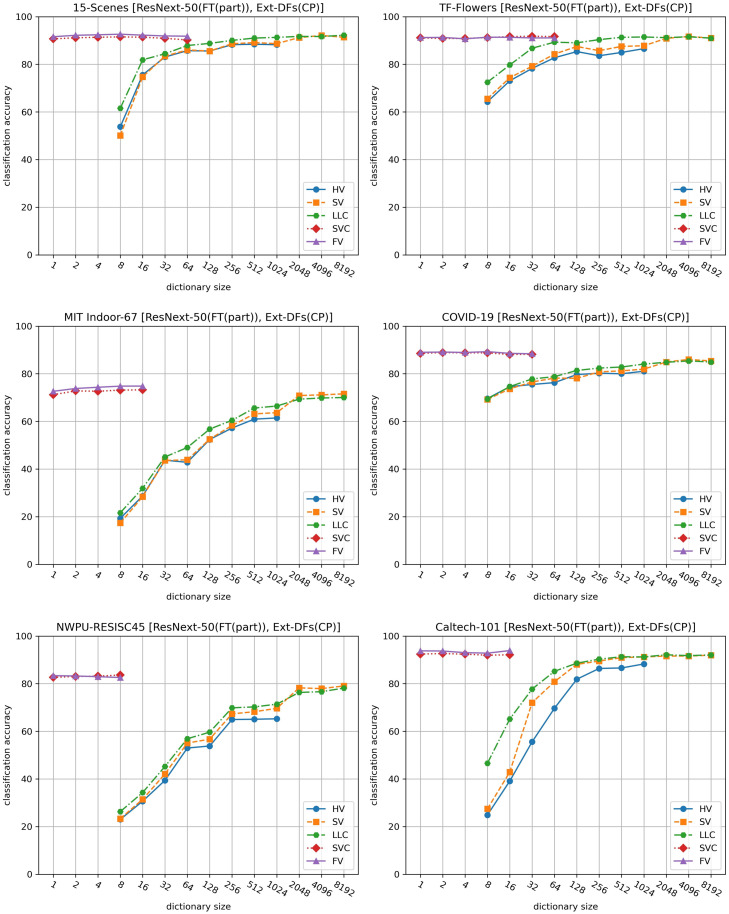
Comparison of HV, SV, LLC, SVC and FV on six datasets.

In this subsection, we investigate the feature encoding method. Fig 2 presents the comparative results of five feature encoding methods, obtained using the average pooling layer of ResNext-50(FT(part)) to generate deep features. It’s important to note that the conclusions drawn here are not exclusive to this model, similar conclusions can also be observed with other DL models.

As shown, FV consistently outperforms the other methods, achieving the highest accuracies across all datasets. This observation aligns with the findings in [11], which suggest that FV’s classification performance excels when encoding deep features.

Interestingly, SVC’s performance is closely comparable to that of FV. Both methods achieve the highest, or near-highest, accuracies when the dictionary size is set to 1. The difference in accuracy between FV and SVC is significantly less than when encoding handcrafted features, as demonstrated in [13]. Furthermore, the dimensionality of coding vectors obtained by SVC is nearly half of that obtained by FV. These findings suggest that SVC could serve as a viable alternative to FV. As such, it would be worthwhile to explore the application of SVC in feature encoding or the design of end-to-end DL models that incorporate the SVC calculation process for image classification tasks.

On the other hand, HV, SV, and LLC underperform when compared to FV and SVC. These methods require a larger dictionary size (e.g., 2048 or larger) to achieve optimal accuracies, which significantly increases computational costs. Among these, LLC slightly outperforms HV and SV in terms of classification accuracy.

**Fig 3:**
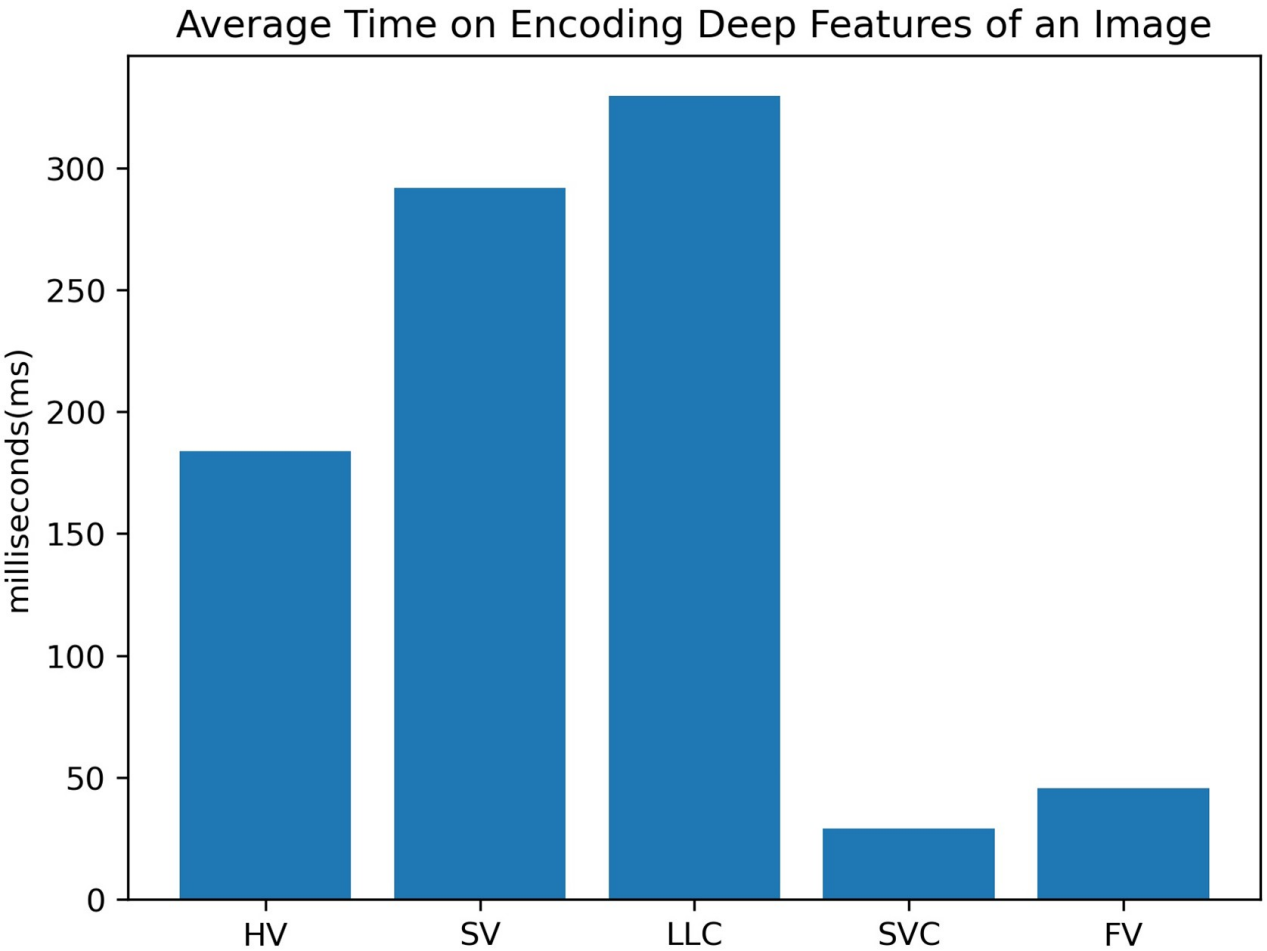
Average time of HV(*K*=1024), SV(*K*=2048), LLC(*K*=2048), SVC(*K*=1) and FV(*K*=1) for encoding deep features of an image.

To further illustrate the efficiency of these coding methods, Fig 3 presents the average time taken by each method to encode deep features of an image, based on the highest accuracy achieved on the Caltech-101 dataset. SVC and FV prove to be much more efficient than HV, SV, and LLC. While SVC’s performance is marginally lower than FV’s by only 0.7% (93% vs. 93.7%), it is one-third faster than FV in our experiments, further highlighting its potential as a practical alternative.

In summary, FV consistently outperforms other methods across all datasets. SVC also demonstrated potential as an effective alternative due to its comparable performance. In our subsequent experiments, FV will be adopted as the default encoding method.

### 4.2 Factor 2: Feature extraction method

**Fig 4:**
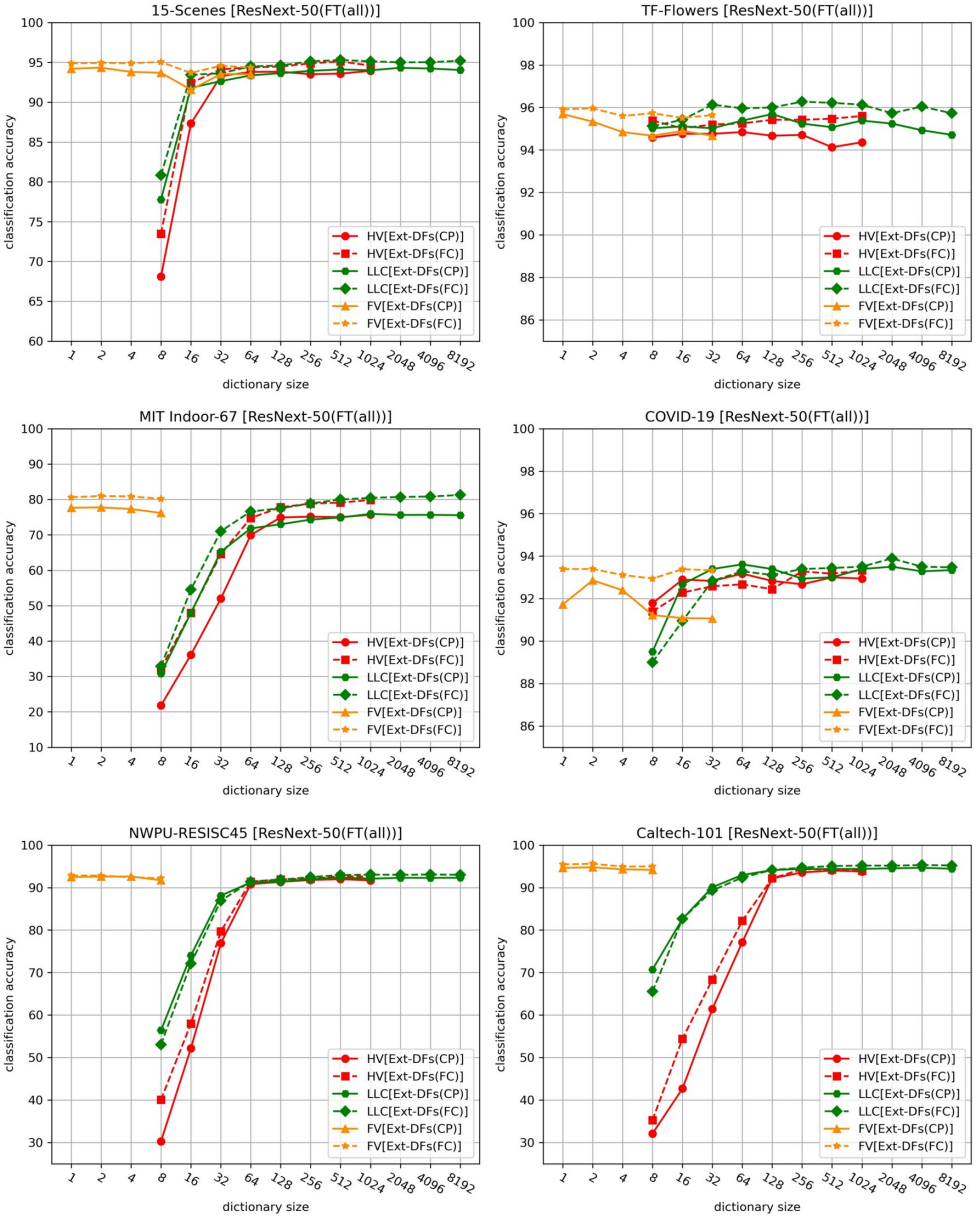
Comparison of two feature extraction methods Ext-DFs(CP) and Ext-DFs(FC) on six datasets.

**Fig 5:**
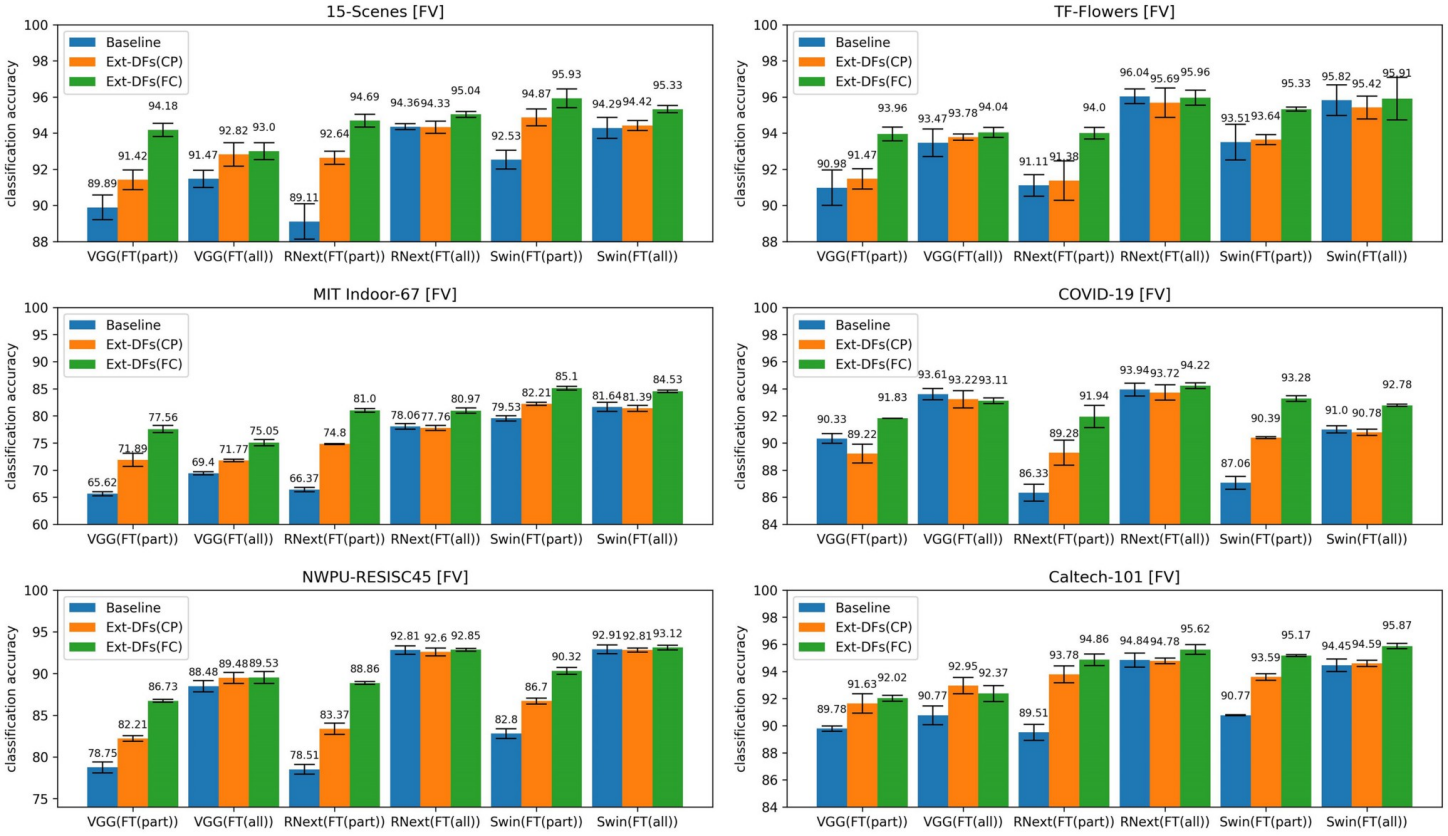
Accuracy comparison of 12 combinations of three pre-trained DL models, two fine-tuning strategies, and two feature extraction methods on six datasets. VGG: VGGNet-16, RNext: ResNext-50, Swin: SwinTransformer.

Another factor we investigated here is the feature extraction method. Fig 4 compares Ext-DFs(CP) and Ext-DFs(FC) under different dictionary sizes using three encoding methods when ResNext-50(FT(all)) is used to generate deep features, and Fig 5 shows the comparison results of these two feature extraction methods with different feature extractors when FV is used.

As shown in Figs 4 and 5, Ext-DFs(FC) outperforms Ext-DFs(CP) in terms of classification accuracy in the majority of cases. However, it’s important to note that the higher accuracy achieved by Ext-DFs(FC) over Ext-DFs(CP) comes at the expense of significantly higher computational costs. Ext-DFs(CP) requires only a single inference of the DL model to obtain all deep features of an image, whereas Ext-DFs(FC) necessitates an inference for each image patch. As per [40], extracting multi-scale image patches can yield higher accuracy than extracting single-scale image patches. In our experiments, we extract image patches of six different scales. For each image, Ext-DFs(FC) can generate about 133 patches, making the extraction costs of Ext-DFs(FC) approximately 133 times that of Ext-DFs(CP).

Moreover, Ext-DFs(FC) does not always surpass Ext-DFs(CP) in terms of classification accuracy. For instance, when the dictionary size is small (e.g., 8, 16, and 32) and LLC is used, Ext-DFs(CP) even outperforms Ext-DFs(FC) on datasets such as COVID-19 CXR, NWPU-RESISC45, and Caltech-101. Additionally, the accuracy improvement offered by Ext-DFs(FC) is negligible on NWPU-RESISC45 when using FV.

In summary, while Ext-DFs(FC) often outperforms Ext-DFs(CP) in terms of classification accuracy, it comes with higher computational costs. Also, its superiority is not consistent across all conditions and datasets. These findings highlight the importance of carefully selecting the feature extraction method based on the specific requirements of the task at hand.

### 4.3 Factor 3: Model architecture

The third factor we investigated here is the model architecture. As shown in Fig 5, the classification accuracy of the BoDVW model generally improves as the DL model becomes more advanced. Even if the accuracy of a more advanced DL model is inferior to a less advanced one, the BoDVW model using the former still achieves positive gains over the latter. For example, while the accuracy of ResNext-50(FT(part)) drops slightly on some datasets compared to VGGNet-16(FT(part)), the BoDVW model using ResNext-50(FT(part)) still achieves gains. When FT(all) is adopted, the improvement trend is less obvious than when FT(part) is used. For instance, the accuracy gain obtained by replacing ResNext-50(FT(part)) with SwinTransformer(FT(part)) on the 15-Scenes dataset is 1.24%, while it decreases to 0.29% when replacing ResNext-50(FT(all)) with SwinTransformer(FT(all)). Notably, there are two exceptions on the TF-Flowers and COVID-19 CXR datasets where the accuracies drop when using the more advanced SwinTransformer(FT(all)). This suggests that when the pre-trained DL model is fine-tuned by FT(all), the performance of the BoDVW model may not necessarily improve with the increasing advancement of the DL model.

### 4.4 Factor 4: Fine-tuning strategy

In this subsection, we investigate the fine-tuning strategy, the fourth factor in our study. As shown in Fig 5, when Ext-DFs(CP) is adopted, the feature extractor fine-tuned by FT(all) achieves higher classification accuracy in 15 out of 18 control groups, compared to the feature extractor obtained by FT(part). When Ext-DFs(FC) is used, FT(all) outperforms FT(part) in 12 out of 18 control groups. This indicates that in most cases, the feature extractor fine-tuned by FT(all) achieves higher classification accuracy than the one fine-tuned by FT(part).

However, using DL models fine-tuned by FT(part) results in larger accuracy gains over the baselines compared to those fine-tuned by FT(all). For instance, when employing ResNext-50(FT(part)), Ext-DFs(CP) and Ext-DFs(FC) achieve accuracy gains of 3.53% and 5.58% on the 15-Scenes dataset, respectively. In contrast, when using ResNext-50(FT(all)), the gains decrease to -0.03% and 0.68%, respectively.

In conclusion, while using pre-trained DL models fine-tuned by FT(all) as feature extractors can yield higher accuracies, using pre-trained DL models fine-tuned by FT(part) tends to result in larger accuracy gains over the baselines.

### 4.5 Factor 5: Number of training samples

**Fig 6:**
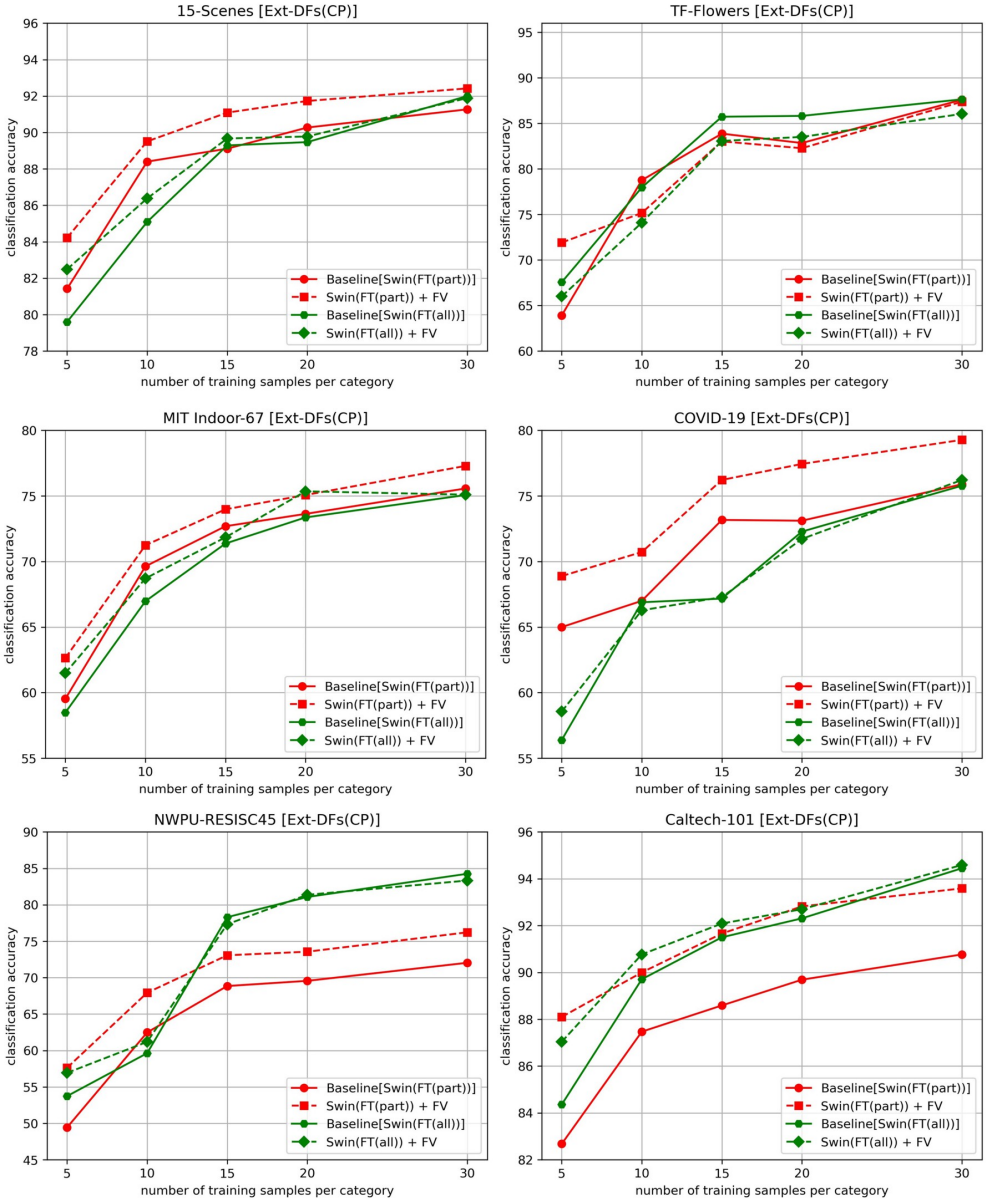
Comparison of the accuracies obtained when the pre-trained SwinTransformer is fine-tuned with different number of training images. Swin: SwinTransformer.

In this subsection, we investigate the accuracy gains brought by using the pre-trained SwinTransformer as a feature extractor when the number of training images (the fifth factor) is insufficient. The pre-trained SwinTransformer is fine-tuned using different numbers of training samples (5, 10, 15, 20, 25 and 30 images per category). Fig 6 shows the experimental results obtained on six datasets when Ext-DFs(CP) is used for feature extraction and FV for feature encoding.

Overall, compared to using SwinTransformer (FT(all)) as the feature extractor, using SwinTransformer (FT(part)) not only yields significantly larger accuracy gains over baselines on 5 out of 6 datasets but also achieves the highest accuracies on the 15-Scenes, MIT Indoor-67, and COVID-19 CXR datasets. Apart from the TF-Flowers dataset, stable accuracy gains are maintained on other datasets as the number of training samples increases. When using SwinTransformer (FT(all)) as the feature extractor, there are no accuracy improvements on the TF-Flowers dataset, and the accuracy gains gradually disappear on other datasets as the number of training samples increases.

This phenomenon can be attributed to the fact that FT(all) fine-tunes the parameters of all layers, which can lead to an overfitting issue when there is a scarcity of training samples. As a result, the knowledge gained from another task can be partially forgotten due to the alteration of parameters across all layers, thereby decreasing the quality of deep features. On the other hand, FT(part) only re-trains the parameters of the classification layer, preventing the loss of previously acquired knowledge.

### 4.6 Overall analysis

In this subsection, the key question of our study is first answered based on the experimental results. Following this, the five factors are in-depth analyzed from the perspective of high-level semantics to understand the experimental results. Finally, the BoDVW model is compared with other works in terms of classification accuracy, and potential strategies to improve its classification performance are stated.

#### 4.6.1 Answer to the key problem of our study

Based on the experimental results presented above, the answer to the central question of this study is negative: the practice of using a pre-trained DL model as the feature extractor in the BoDVW model does not always improve image classification accuracy. In the hyperparameter space constituted by the five factors investigated above, there exist some parameter combinations where this practice does not result in accuracy gains. Especially, with FT(all) and Ext-DFs(CP), this practice can easily lead to a decrease in accuracy. For instance, when the model is ResNext-50(FT(all)), this practice resulted in an accuracy drop across all datasets.

However, we also found that many parameter combinations within certain hyperparameter subspaces can lead to accuracy gains over baselines. When there are sufficient training images for fine-tuning and FV is used for feature encoding, significant accuracy improvements are achieved on all datasets when using the pre-trained DL model fine-tuned by FT(part). The only exception occurs when the dataset is COVID-19 CXR, the feature extraction method is Ext-DFs(CP), and the model is VGGNet-16. Apart from this case, the accuracy improves by 0.13% to 8.43% (averaged at 3.11%) with Ext-DFs(CP) and by 1.06% to 14.63% (averaged at 5.66%) with Ext-DFs(FC). When using the pre-trained DL model fine-tuned by FT(all), the accuracy is improved in 14 out of 18 experiments by 0.21% to 5.65% (averaged at 1.58%) with Ext-DFs(FC). The decrease in accuracy occurs on the TF-Flowers and COVID-19 CXR datasets. At this time, accuracy gains were not observed with all pre-trained DL models. In contrast, accuracy gains were only obtained in 7 out of 18 experiments with Ext-DFs(CP). When there are insufficient training images for fine-tuning, it is not recommended to use pre-trained DL models fine-tuned by FT(all) as feature extractors. Instead, using pre-trained DL models fine-tuned by FT(part) as feature extractors can yield stable accuracy gains over baselines with a high probability (on 5 out of 6 datasets).

In summary, while the practice of using a pre-trained DL model as the feature extractor in the BoDVW model does not improve accuracy under all conditions, it remains a technique with significant potential for enhancing image classification accuracy.

#### 4.6.2 In-depth analysis of the five factors

In the context of using pre-trained DL models to solve new image classification tasks with insufficient training images, there is potential to obtain higher classification accuracy by employing a pre-trained DL model as the feature extractor, compared to directly using the new classification layer of the model for image classification. The reason is that, compared to the single image feature representation vector input to the model’s classification layer, the set of deep features, converted from different image patches, more explicitly contain both local and global high-level semantic information of the image. When Ext-DFs(FC) is used, deep features are converted from image patches of different scales and posistions. When Ext-DFs(CP) is employed, the receptive fields of deep features are same in scale but different in focus point. Thus deep features can be viewed as being converted from image patches of the same scale at different positions. The classification performance of the BoDVW model is influenced by mutiple aspects.

1. **Accuracy of deep features in expressing the high-level semantic information of image patches:** If deep features cannot accurately represent the high-level semantics of image patches, then the deep features of image patches with the same semantics are not adjacent in the feature space. This could result in the coding vectors of these image patches being different, thereby impairing the discriminability of the final image representation vector. There are three factors closely related to the accuracy of deep features in expressing the high-level semantic information of image patches.
  The first factor involves fine-tuning the pre-trained DL model. When the pre-trained DL model is fine-tuned by FT(part) with the new task data (only the new classification layer is re-trained), it will lead to a less accurate description of the high-level semantic information of images from the task, limiting its classification performance. However, this problem can be alleviated by utilizing the set of deep features that express the high-level semantic information of different image patches. Once the entire model is fine-tuned, the description of images’ high-level semantic information becomes more accurate, significantly reducing the gap between the single image feature representation vector and the set of deep features in expressing images’ high-level semantic information. Hence, the accuracy gains obtained with FT(part) are larger than the gains with FT(all), as shown in Fig 5.
  The second factor involves the model architecture. As shown in Fig 5, when using pre-trained DL models as feature extractors, the accuracies obtained by SwinTransformer are higher than those by other DL models in most cases. This is because SwinTransformer can better capture the high-level semantic information than ResNext-50 and VGGNet-16 due to its more advanced network architecture. Specifically, when FT(part) is used, the capability of SwinTransformer in capturing high-level semantic information is obviously stronger than other DL models. This is supported by the fact that it performs better than other DL models in accuracy when directly using their new classification layers for classification. When FT(all) is employed, the capability of all DL models in capturing high-level semantic information improves. Especially, the gap between ResNext-50 and SwinTransformer is significantly reduced, leading to very close accuracies when using them as feature extractors. The significant exception appears on the COVID-19 CXR dataset when FT(all) is adopted. For the COVID-19 CXR dataset, the categorization of its images is largely determined by mid-level semantic information. Among the three deep learning models VGGNet-16, ResNext-50, and SwinTransformer, VGGNet-16 is relatively less capable of capturing high-level semantic information compared to the other two models. ResNext-50 can capture mid-level semantic information due to its stacked residual modules that allow low-level or mid-level features to be directly passed to higher levels. However, SwinTransformer only uses residual connections in each Transformer block’s self-attention sub-layer and feed-forward neural network sub-layer, making it less effective than ResNext-50 at capturing mid-level semantic information. As a result, using SwinTransformer(FT(all)) as a feature extractor actually resulted in lower classification accuracies than VGGNet-16(FT(all)) and ResNext-50(FT(all)).
  Besides these two factors, the number of training samples (the third factor) also plays a crucial role when using FT(all). If there are insufficient training images for fine-tuning the entire DL model, an overfitting problem could occur, thereby failing to capture the high-level semantic information of images.
2. **Image patches’ semantic information:** As stated above, the effectiveness of the BoDVW model lies in its ability to more explicitly describe the local and global high-level semantic information of an image based on different image patches extracted from it. Compared to Ext-DFs(CP), Ext-DFs(FC) can extract more diverse image patches, leading to superior classification accuracy. Especially when the pre-trained DL model is fine-tuned by FT(all) and used as the feature extractor, the deep features with the same receptive field size obtained by Ext-DFs(CP) cannot provide more semantic information than the single image representation vector input to the model’s classification layer. As a result, it does not result in higher classification accuracies than the baselines when ResNext-50 and SwinTransformer are used. As for VGGNet-16, since its less advanced architecture is not enough to accurately capture the high-level semantic information, there is still room for the set of deep features to provide more semantic information. In contrast, Ext-DFs(FC) almost always leads to accuracy gains because the multi-scale image patches extracted by it not only contain the semantic information of local image regions (e.g., 96×96 pixels), but also the information of global image regions (e.g., 256×256 pixels).
3. **Accuracy of coding vectors in representing deep features:** In our experiments, we used five feature encoding methods—HV, SV, LLC, SVC, and FV. The coding vectors obtained by these methods for the same deep feature show increasing accuracy in representing it. HV uses only one visual word to roughly represent a deep feature. Both SV and LLC use multiple visual words to encode a feature. SVC primarily captures first-order statistical information, without considering the shape of the feature distribution or the degree of variation. In contrast, FV captures not only first-order statistical information (mean), but also second-order statistical information, which includes the variance or shape of the feature distribution. This allows FV to provide a richer and more detailed feature representation. As demonstrated in our experiments, FV leads to higher classification accuracy than the other encoding methods in almost all cases.

#### 4.6.3 Classification performance of the BoDVW model

We compared the highest accuracies obtained by the BoDVW model on the six datasets with those of other works, as shown in Table 5. The BoDVW model slightly underperforms the highest-performing methods on four of the six datasets, with a difference of 0.05% to 1.47%. On the MIT Indoor-67 dataset, the highest accuracy of 94.1% is obtained by FTOTLM Aug. This method achieves a significant accuracy improvement, from 74.6% to 94.1%, by applying a special data augmentation technology tailored for scene recognition. This technology could potentially be used to improve pre-trained DL models when using them as the feature extractor. Excluding this work, the BoDVW model underperforms the highest-performing method by 3.33%. On the less commonly used COVID-19 CXR dataset, the BoDVW model achieves an accuracy improvement of 6.36% over the work of C.W. Lin et al. Overall, the BoDVW model demonstrates comparable accuracy to other works across the six datasets.

**Table 5:** Comparison with other recent works on 15-Scenes(15S), TF-Flowers(TFF), NWPU-RESISC45(NWPU), MIT Indoor-67(MIT), COVID-19 CXR (COV), and Caltech-101(CAL). A: SwinTransformer(FT(part)) + Ext-DFs(FC) + FV, B: ResNext-50(FT(all)) + Ext-DFs(FC) + FV, C: SwinTransformer(FT(all)) + Ext-DFs(FC) + FV.

Method	Year	15S	TFF	NWPU	MIT	COV	CAL
BoDVW model	-	95.93 ± 0.52^*A*^	95.96 ±0.41^*B*^	93.12 ±0.28^*C*^	85.1 ±0.35^*A*^	**94.22 ± 0.21** ^*B*^	95.87 ±0.19^*C*^
Places365-VGGNet [52]	2018	91.97	-	-	-	-	-
MVBOW [53]	2021	92.9	-	-	-	-	-
FTOTLM no Aug. [19]	2019	94	-	-	74.6	-	-
DUCA [39]	2016	94.5	-	-	71.8	-	-
FTOTLM Aug. [19]	2019	**97.4**	-	-	**94.1**	-	-
M2M BiLSTM [54]	2019	96.3	-	-	88.25	-	-
DFF + ADML [55]	2020	96.39	-	-	88.43	-	-
M. Saini [12]	2021	-	88.16 ± 0.84	-	-	-	-
TUNELOSS [56]	2019	-	90.43	-	-	-	80.7
S. Giraddi [57]	2020	-	95	-	-	-	-
R. Murugeswari [58]	2022	-	**97**	-	-	-	-
VGGNet-16 + BoCF [24]	2017	-	-	84.32	-	-	-
CNN-SC [59]	2019	-	-	85.29	-	-	-
VGG_VD16 + SAFF [60]	2021	-	-	87.86 ± 0.14	-	-	-
ResNet-50 + EAM [61]	2021	-	-	93.51 ± 0.12	-	-	-
ResNet-101 + EAM [61]	2021	-	-	**94.29 ± 0.09**	-	-	-
LLC [62]	2019	-	-	-	79.63	-	-
S. Xu [63]	2021	-	-	-	80.22	-	-
FV-CNN [64]	2015	-	-	-	81	-	-
C.W. Lin [65]	2022	-	-	-	87.59	-	-
C. Sitaula [25]	2021	-	-	-	-	87.86	-
M. Bansal [66]	2021	-	-	-	-	-	93.73
AutoFCL [67]	2021	-	-	-	-	-	94.38
N.K. Singh [68]	2020	-	-	-	83.6	-	94.2
AutoTune [69]	2021	-	-	-	-	-	**95.92 ± 0.025**

Although the classification accuracies obtained by the BoDVW model are not the highest, a number of research techniques from the BoVW model could potentially be applied to improve the classification performance of the BoDVW model. Specifically, it can be improved in the following ways: One approach is to enhance the feature extractor. This can be achieved through various methods such as data augmentation, careful fine-tuning, and the use of more advanced model architectures. Another way is to improve the stages of dictionary learning, feature encoding, and feature pooling. For instance, the method proposed in [51] can be employed to learn a discriminative dictionary, which could significantly enhance the model’s ability to distinguish between different classes. Furthermore, the pooling methods introduced in [26, 27] could be used to aggregate the coding vectors of deep features, potentially enhancing the model’s performance by creating more robust feature representations.

In addition to adopting these existing techniques, the BoDVW model could potentially achieve further performance improvements by considering the following two aspects.

Firstly, the workflow of the BoDVW model is derived from that of the BoVW model. However, this workflow is designed for low-level semantic features (e.g., SIFT), and it completely ignores the high-level semantic characteristics of deep features. For instance, one high-level semantic characteristic of deep features is that there are many local regions in the feature space where deep features primarily originate from one or a few categories. This is because the high-level semantic information of an image patch is generally closely related to the image category and typically exists in one or a few categories. In such cases, deep features converted from image patches with the same semantics are not only close to each other in the feature space but also primarily originate from one or a few categories. This feature distribution characteristic is quite different from that of low-level semantic features.

Secondly, while Ext-DFs(FC) can achieve higher classification accuracy than Ext-DFs(CP), it requires tens of times the feature extraction cost of the latter. Considering the different receptive field sizes of different layers in DL models, an ideal approach is to extract high-level semantic features from smaller receptive fields. However, it’s important to note that the features extracted directly from shallow layers with smaller receptive fields lack sufficient semantic information.

## 5 Conclusion

In this study, we dedicated ourselves to a key question, namely, can the use of a pre-trained DL model as the feature extractor in the BoDVW model always improves image classification accuracy? To answer this question, we investigated several factors related to the feature extractor, including model architecture, fine-tuning strategy, number of training samples, feature extraction method, and feature encoding method. Common settings are chosen for each factor. These factors constitute a hyperparameter space. We answered this key question based on this hyperparameter space.

The experimental results on six datasets confirmed that the answer to this question is negative. In this hyperparameter space, there are some parameter combinations where no accuracy gain is obtained. However, within certain hyperparameter subspaces, accuracy gains can always or in most cases be achieved. Under the premise of using FV for feature encoding, when using the pre-trained DL model fine-tuned by FT(part), significant accuracy improvements can be achieved with a high probability (35 out of 36 experiments in this article). When using the pre-trained DL model fine-tuned by FT(all), accuracy gains can be obtained in most cases (14 out of 18 experiments in this article) with Ext-DFs(FC).

The research in this work has proven that the practice of using a pre-trained DL model as the feature extractor in the BoDVW model is a technique with significant potential for accuracy improvement.
